# *PPAN* modulates mouse male germ cell development via maintaining nucleolar homeostasis

**DOI:** 10.1016/j.gendis.2024.101204

**Published:** 2024-01-03

**Authors:** Yu Tian, Yufan Wang, Qing Tian, Guiping Cheng, Li-quan Zhou

**Affiliations:** a Institute of Reproductive Health, Tongji Medical College, Huazhong University of Science and Technology, Wuhan, Hubei 430030, China; b Department of Gynecology and Obstetrics, Zhongnan Hospital of Wuhan University, Wuhan, Hubei 430030, China

In mammalian cells, the nucleolus serves as a factory for organizing ribosomal RNA (rRNA) synthesis and ribosomal biogenesis. Moreover, by interacting with rRNA genes and heterochromatin, nucleolus also functions in chromatin organization and reprogramming. Differing from somatic cells, nucleolus undergoes distinct morphological changes during oocyte and early embryo development, while playing an indispensable role in orchestrating chromatin remodeling.^1^ However, the structural change and function of nucleolus in male germ cell development are still waiting for exploration. We recently reported that the role of *Peter Pan Homolog* (*PPAN*), a key rRNA processing factor, in mouse oocyte and preimplantation development.^2^ Here, we further constructed a male germ cell-conditional *PPAN* knockout (*PPAN*-cKO) mouse model to disturb nucleolar function in spermatogenesis. Our data showed that depletion of *PPAN* severely disrupts germ cell development by inducing aberrant nucleolar structure and function, resulting in an oligoasthenoteratozoospermia phenotype in mice.

We first evaluated the expression pattern of *PPAN* in mouse tissues and testicular cells and found that *PPAN* was highly expressed in the testes, especially in spermatogonia (Fig. S1A–D). Subsequently, we generated *PPAN*-cKO mice using Stra8^Cre^ to delete exons 1–11 of *PPAN* gene specifically in differentiated spermatogonia (Fig. S2A; Fig. 1A). *PPAN*-cKO mice were identified by genotyping analysis (Fig. S2B) and significantly reduced *PPAN* expression was confirmed compared with controls at the transcription and translation levels (Fig. 1B; Fig. S2C). *PPAN*-cKO mice showed smaller litters and decreased fertilization rate detected by *in vitro* fertilization experiment (Fig. 1D; Fig. S3). Phenotypic analysis suggested that *PPAN*-cKO adult mice exhibited obvious testicular atrophy, reduced weight ratios of testis and epididymis to the body, and lower sperm counts compared with controls (Fig. 1C–E). Further histological analysis indicated that seminiferous tubules were significantly atrophic and epididymis had less spermatozoa in *PPAN*-cKO (Fig. 1F). Additionally, *PPAN*-cKO had an increased rate of abnormal sperm and significantly decreased sperm motility compared with control mice. The main types of abnormal sperms in *PPAN*-cKO were folded tail and coiled tail, which could be crucial factors leading to the severe decline in sperm motility (Fig. 1G–I). Furthermore, *PPAN*-cKO sperms had an elevated proportion of acrosomal hypoplasia and a slight reduction in mitochondrial intensity (Fig. S4).

**Figure 1:**
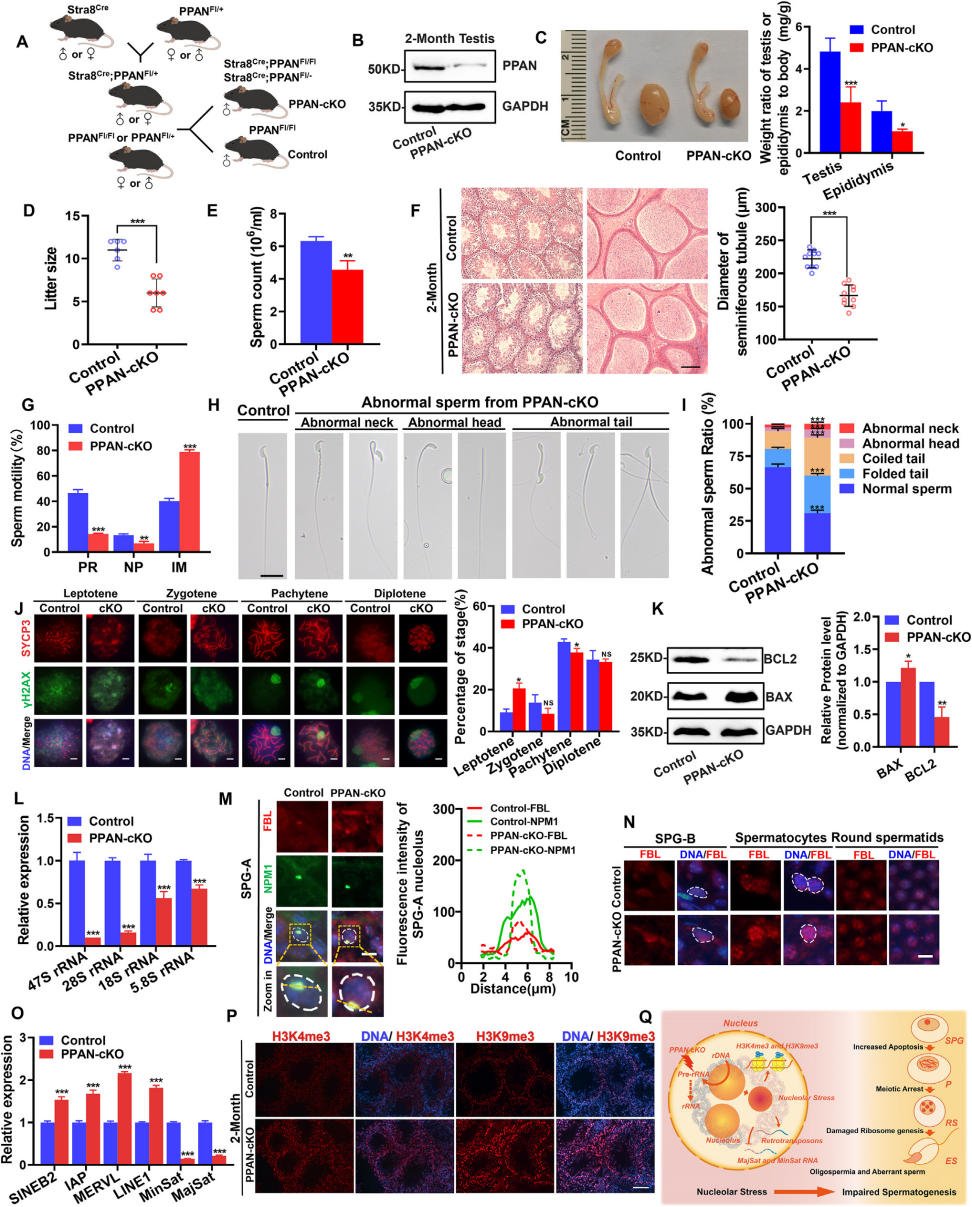
*PPAN* deficiency disturbs male germ cell development. **(A)** Schematic illustration of a breeding strategy for *PPAN*-cKO mice. **(B)** The expression of PPAN in the testes of *PPAN*-cKO mice analyzed by Western blot. **(C)** Gross morphology of the testis and epididymis from adult control and *PPAN*-cKO mice, and relative weight ratio to body analysis (*n* = 4). **(D)** Pup number per litter from adult control and *PPAN*-cKO mice. **(E)** Sperm count of adult control and *PPAN*-cKO mice (*n* = 3). **(F)** Hematoxylin and eosin staining of testis sections from two-month-old control and *PPAN*-cKO mice (Scale bar = 100 μm) and quantitative comparison of seminiferous tubule diameter. **(G)** Sperm motility of adult control and *PPAN*-cKO mice (*n* = 3). PR, progressive motility; NP, non-progressive motility; IM, immotility. **(H)** Representative sperm morphology of adult control and *PPAN*-cKO mice, including normal sperm, twisted neck, bent neck, round head, headless, folded tail, coiled tail, and double tails (Scale bar = 10 μm). **(I)** Comparison of the proportion of abnormal sperm between the control and *PPAN*-cKO mice (*n* = 3). More than 200 sperms were counted from three different mice in each group. **(J)** Meiotic spermatocyte spreads for three-week-old control and PPAN-cKO mice. Co-immunostaining of SYCP3, γH2AX, and DAPI (Scale bar = 10 μm), and percentage of each meiotic stage in control and PPAN-cKO spermatocytes. 500–800 spermatocytes were counted from three different mice in each group. **(K)** Western blot analysis of proteins associated with apoptosis pathways in control and *PPAN*-cKO testes on post-natal day 10 and quantitative analysis (*n* = 3). **(L)** Expression changes of rRNA genes in the testes of control and PPAN-cKO mice on post-natal day 7 determined by reverse transcription quantitative PCR. **(M)** Co-immunostaining of FBL and NPM1 in control and PPAN-cKO SPG-A (type-A spermatogonia), and distribution and intensity analyses on nucleolus fluorescence along the longest axis (marked by yellow line) of the nucleolus using ImageJ (DNA was stained with Hoechst, scale bar = 10 μm). **(N)** Expression and location analyses of FBL in control and PPAN-cKO SPG-B (type-B spermatogonia), spermatocytes, and round spermatids (DNA was stained with Hoechst, scale bar = 10 μm). **(O)** Expression changes of retrotransposon genes in two-month-old control and PPAN-cKO mice determined by reverse transcription quantitative PCR. **(P)** Immunostaining of H3K4me3 and H3K9me3 in sections of the control and *PPAN*-cKO testes (DNA was stained with Hoechst, scale bar = 100 μm). **(Q)** Schematic illustration of how *PPAN* deficiency impairs male germ cell development.

To explore the reasons behind the smaller testes and lower sperm counts observed in the *PPAN*-cKO mice, we employed immunostaining on seminiferous tubule sections to examine two marker proteins, DDX4 (a marker of germ cells) and Ki67 (a marker of actively proliferating cells). Our results showed that the intensity of DDX4 was decreased from the spermatogonia stage and a significantly reduced Ki67 intensity in *PPAN*-cKO mice, indicating that the spermatogonia proliferative activity of spermatogonium of cKO mice was damaged (Fig. S5A, B). Besides, TUNEL staining showed increased apoptotic spermatogonia and spermatocytes in the seminiferous tubules of *PPAN*-cKO mice (Fig. S5C). Subsequently, we examined the progression of meiosis I metaphase. Immunostaining analysis of γH2AX (a marker of DNA double-strand break) and PAS staining suggested an increased number of leptotene and zygotene spermatocytes in the seminiferous tubules of adult *PPAN*-cKO mice compared with controls, indicating a potential arrest in meiosis (Fig. S6). Then we employed chromosomal spreads of spermatocytes via immunostaining using antibodies against SYCP3 (a marker of synaptonemal complex) and γH2AX. The results showed that there was a higher proportion of leptotene-stage spermatocytes and a lower proportion of zygotene spermatocytes in *PPAN*-cKO mice compared with controls, indicating a delay in the transition from leptotene to later stages (Fig. 1J). PAS staining on testes of two-month-old mice for analyzing seminiferous epithelium also confirmed this result (Fig. S7). Besides, we detected alterations of protein expression of apoptosis-related factors in the testis on post-natal day 10 using Western blot assay, characterized by increased BAX, decreased BCL2, and increased Caspase3 (Fig. 1K; Fig. S8). PPAN plays a key role in maintaining the stability of pre-ribosomal RNA (pre-rRNA). Our result showed that the levels of 47 S pre-rRNA were significantly suppressed following *PPAN* deficiency (Fig. 1L). Interestingly, pre-rRNA has been linked to the formation of the XY body during meiotic prophase,^3^ which could explain the meiotic arrest in *PPAN*-cKO mice. Overall, depletion of *PPAN* resulted in defects in the proliferation of spermatogonia and delay in meiosis I progress of spermatocytes, which further contributed to reduced sperm production.

Regarding the high proportion of abnormal sperm in *PPAN*-cKO mice, we speculate that this may be attributed to *PPAN* deficiency-induced abnormal ribosome biogenesis, according to a previous study of the role of ribosomes in regulating sperm motility.^4^ In the testis on post-natal day 7 (late spermatogonial stage), we observed a significant inhibition of multiple mature rRNA levels (5.8 S rRNA, 18 S rRNA, and 28 S rRNA), as well as abnormal expression of various nucleolar factors in *PPAN*-cKO compared with controls, suggesting dysfunction in the nucleolus (Fig. 1L; Fig. S9A). The typical nucleolar organization consists of three distinct layers, referred to as the fibrillar center, dense fibrillar component, and granular component.^1^ We performed immunostaining using anti-FBL (fibrillarin, marker of dense fibrillar component) and anti-NPM1 (nucleophosmin1, marker of granular component) antibodies to label nucleolus. Sertoli cells with intact PPAN expression exhibited normal nucleolar structure (Fig. S9B). In the type-A spermatogonia of *PPAN*-cKO, nucleolus labeled with FBL and NPM1 showed a smaller and more condensed structure compared with the control, and there was mildly increased dispersion of FBL localization in the nucleoplasm (Fig. 1M; Fig. S9C, D). These results indicate that *PPAN* deficiency leads to impaired rRNA synthesis and subsequent nucleolar stress response.

Another attractive concern is how nucleolar structure changes during spermiogenesis. Our results showed that typical nucleolar structure began to disintegrate during the type-B spermatogonia stage, and FBL and NPM1 were dispersed in the nucleus in the form of protein clusters in both spermatocytes and round spermatids (Fig. 1N; Fig. S9E). These findings firstly suggested the structural changes of nucleolus during spermatogenesis. *PPAN* depletion also affected the dynamic changes of nucleolar structure, as evidenced by abnormal up-regulation and aggregation of FBL in the spermatocytes and round spermatids (Fig. 1N). Our previous study has confirmed that the *PPAN* lacks chromatin binding activity,^2^ however, whether *PPAN* participates in epigenetic regulation via inducing nucleolar stress remains an intriguing question. We measured the expression of retrotransposons in two-month-old control and *PPAN*-cKO testes, and the results showed that transposons including SINEB2, LINE1, and IAP were slightly up-regulated, and the expression of Minor Satellite (MajSat) and Major Satellite (MajSat) was markedly suppressed (Fig. 1O). This could be due to the repression of rRNA expression resulting from the absence of *PPAN*.^5^ We also detected the levels of multiple histone modifications in two-month-old control and *PPAN*-cKO testes (Fig. 1P; Fig. S10). Intriguingly, *PPAN* deficiency simultaneously facilitated the abundance of activating modification H3K4me3 and repressive modification H3K9me2 and H3K9me3. Collectively, these results indicated that *PPAN* deficiency results in alterations in nucleolar-associated chromatin landscape, which could potentially have sustained effects on germ cell development (Fig. 1Q).

Taken together, we demonstrated that *PPAN* deficiency induced nucleolar stress in spermatogonium, disrupting ribosome biogenesis and affecting the chromatin environment, collectively impairing germ cell development. In addition, we first uncovered the patterns of nucleolar structural alterations during mammalian spermatogenesis and highlighted their association with chromatin remodeling in this process. In summary, we identified that *PPAN* regulates germ cell development by maintaining nucleolar homeostasis and proposed that *PPAN* mutation should be considered a driver contributing to oligoasthenoteratozoospermia.

## Ethics declaration

The animal procedures were approved by the Institutional Animal Care and Use Committee of Tongji Medical College, Huazhong University of Science and Technology. Mice were housed in the specific pathogen-free facility at Huazhong University of Science and Technology. All animal experiments were conducted in accordance with ethical guidelines outlined in the Guide for the Care and Use of Laboratory Animals.

## Author contributions

L.Z. designed and supervised this study. Y.T., Y.W., Q.T., and G.C. carried out the experiments. Y.T. and Y.W. analyzed the data and wrote the manuscript. L.Z. reviewed and edited the final manuscript. All authors contributed to the article and approved the submitted version.

## Funding

This work was supported by the 10.13039/501100001809National Natural Science Foundation of China (No. NSFC 32170820), the National Key R&D Program of China (No. 2022YFC2702704), and the Program for HUST Academic Frontier Youth Team.

## Conflict of interests

The authors declare no conflict of interests.
