# Supplementary material for: PPAN modulates mouse male germ cell development via maintaining nucleolar homeostasis
Source: Genes Dis. 2024 Jan 3;12(1):101204. doi: 10.1016/j.gendis.2024.101204 (PMC11462204; doi:10.1016/j.gendis.2024.101204)
Supplement: Multimedia component 1 [file mmc1.docx]

**Supporting Information**

**Materials and methods**

**Ethics statement**

The animal procedures were approved by the Institutional Animal Care and Use Committee of Tongji Medical College, Huazhong University of Science and Technology. Mice were housed in the specific pathogen-free facility at Huazhong University of Science and Technology. All animal experiments were conducted in accordance with ethical guidelines outlined in the Guide for the Care and Use of Laboratory Animals.

**Mice**

Floxed PPAN (PPAN^+/FL^) mice were constructed by microinjection of Cas9, sgRNA, and donor vector into fertilized eggs of C57BL/6 J mice and maintained in a mixed background of C57BL/6 and Chinese Kun Ming strains. Stra8^Cre^ transgenic mice in the C57BL/6J background was purchased from the Jackson Laboratory, and for some unknown reasons, the Stra8^Cre^ is expressed only in the male germ cells in this mouse strain. Stra8^Cre^ transgenic mice were crossed with PPAN^+/FL^ mice to obtain the Stra8^Cre^ PPAN^+/FL^ mice, then the Stra8^Cre^ PPAN^+/FL^ mice were crossed with PPAN^+/FL^ or PPAN^FL/FL^ mice to obtain the Stra8^Cre^PPAN^FL/FL^ and Stra8^Cre^PPAN^FL/-^ (designated as PPAN-cKO) males (Figure 1A). Genotyping was performed by PCR with tail genomic DNA, and genotyping PCR primers are listed in Table S1.

**Antibodies**

The following antibodies were purchased from respective companies: Anti-PPAN (Proteintech, 11006-1-AP), anti-DDX4 (Abcam, ab13840), anti-Ki67 (ABclonal, A11390), anti-SYCP3 (Abcam, ab15093), anti-γH2A.X (Santa Cruz, sc-517348), anti-Fibrillarin (Proteintech, 16021-1-AP), anti-NPM1 (Proteintech, 60096-1-Ig), anti-Bax (Proteintech, 50599-2-Ig), anti-Caspase3 (Proteintech, 19677-1-AP), anti-Caspase8 (Proteintech, 13423-1-AP), anti-Caspase9 (Proteintech, 66169-1-Ig), anti-H3K4me1 (ABclonal, A22078), anti-H3K4me2 (ABclonal, A2356), anti-H3K4me3 (ABclonal, A22146), anti-H3K9me2 (ABclonal, A2359), anti-H3K9me3 (ABclonal, A2360), anti-H3K27me3 (ABclonal, A2363), GAPDH (Proteintech, 60004-1-Ig). Secondary antibodies Goat Anti-Mouse IgG (H+L) CoraLite488 (Proteintech, SA00013-1) and Goat Anti-Rabbit IgG (H+L) CoraLite594 (Proteintech, SA00013-4) were obtained from Proteintech. (1:5,000; A11001) were obtained from Proteintech.

**Histological analysis**

Mouse testes and epididymis were collected and fixed in Bouin’s solution (Phygene, PH0976) at 4°C overnight and then washed with 75% alcohol five times, 30 min each time. Samples were then embedded in paraffin; 5 μm sections were cut and stained with periodic acid–Schiff (PAS) or hematoxylin and eosin (H&E) after being dewaxed and rehydrated. Images were captured by Olympus BX51 Microscope with MShot MSX2 camera.

**Sperm count and motility assays**

For count, the cauda epididymis was collected from 2-month-old mice and incubated in 1 ml PBS at 37°C for 30 min. The sperm suspension was then diluted 1:1 with 0.5 ml 4% PFA and counted using a hemocytometer. For sperm motility assays, sperm were released from the cauda epididymis of 2-month-old mice into 1 ml of pre-warmed F-10 medium (Sigma, USA) and incubated for 15 min at 37°C. Then, 10μl of the sperm suspension were added to a microscope slide and covered with a cover slip for assessment of sperm motility under a microscope.

**Assessment of fertility**

To evaluate the fertility of *PPAN*-cKO mice, 2-month-old *PPAN*-cKO and control mice were bred with two wildtype female mice for at least 4 months. The number of offspring from each pregnant female was documented.

**Western blot analysis**

Mouse testes were collected and proteins were extracted using RIPA buffer containing protease and phosphatase inhibitors (Servicebio, G2002, G2006, and G2008). Equal amounts of protein were separated using SDS-PAGE gel and then transferred to PVDF membranes. Subsequently, the membranes were blocked in a 5% non-fat milk solution for 2 hours(h) at room temperature (RT) and incubated with primary antibodies overnight at 4°C. Following three washes with TBST, the membranes were incubated with a secondary antibody for 2h at RT. Finally, after washing for another three times, the membranes were treated with ECL Western Blotting Substrate (Affinity Biosciences, Cat# KF8001) and detected using ChemiDoc™ XRS+ image system (Bio-Rad). The expression levels of each gene were normalized to GAPDH as an internal standard.

**Immunofluorescence**

Mouse testes were fixed overnight at 4°C in 4% paraformaldehyde, followed by sequential immersion in sucrose solutions with concentrations of 5%, 10%, 12.5%, 15%, and 20%. They were embedded in Tissue-Tek O.C.T. compound (Sakura Finetek, 4583) and rapidly frozen using liquid nitrogen, and then stored at −80°C. Five-micrometer-thick cryosections were cut and washed with PBS three times. Thereafter, for antigen retrieval, cryo-sections were microwaved in 0.01 M sodium citrate buffer (pH 6.0) and then allowed to cool to room temperature. The sections were permeabilized with 0.5% Triton-X100 and incubated with blocking solution (1% Bovine Serum Albumin, Biosharp, BS114) for 1 h to block non-specific antibody binding sites. Subsequently, tissue sections were incubated with primary antibodies overnight at 4°C. Following three washes with PBS, tissue sections were incubated with secondary antibody for 2 h at RT and stained with Hoechst (Beyotime, C1011) or DAPI (Beyotime, C1002). Fluorescence was captured using a FluoView 1000 microscope (Olympus, Japan) equipped with a digital camera (MSX2, Micro-shot Technology Limited, China).

**TUNEL Staining**

Cryo-sections TUNEL staining was performed using TUNEL BrightGreen Apoptosis Detection Kit (A112, Vazyme) according to the manufacturer's procedure. Fluorescence was captured using a FluoView 1000 microscope (Olympus, Japan) equipped with a digital camera (MSX2, Micro-shot Technology Limited, China).

**Active Mitochondrial and PNA Co-immunostaining**

Mouse sperms were incubated with Mito Tracker Red CMXRos (Beyotime, C1032) at 37 °C in 5% CO2 for 30 min. 20μl sperm suspension was dropped on an adhesion microscope slide which was air dried at RT, and then fixed with 4% paraformaldehyde for 30min at RT. Following three washes with PBS, tissue sections were incubated with FITC-PNA (MKBio, MP6327) overnight at 4°C. After washing for another three times, slides were stained with Hoechst (Beyotime, C1011). Fluorescence was captured using a FluoView 1000 microscope (Olympus, Japan) equipped with a digital camera (MSX2, Micro-shot Technology Limited, China).

**Spermatocyte chromosome spreading**

Testes collected from 3-week-old mice were decapsulated and chopped, incubating with 100 mM sucrose solution for 1h. 30μl testicular suspension was dropped on an adhesion microscope slide and mixed with 100μl fixative solution (1% paraformaldehyde ,0.15% Triton X-100 and 0.3 mM NaBorate (pH 7.4). Tilt the slide to spread the cells then let them sit in a humid chamber for 1h. Slides were air dried at RT and stored at −80°C.

**RNA isolation and quantitative RT-PCR**

Total RNAs were extracted from mouse tissues using TRIzol reagent (Invitrogen,15596018) following the manufacturer’s procedure. The purity and concentration of RNA were determined using NanoDrop2000 (Thermo Scientific). cDNA was synthesized using Hifair® Ⅱ 1st Strand cDNA Synthesis Kit (Yeason, 11119ES60). quantitative RT-PCR (RT-qPCR) was performed with SYBR green master mix (Yeason, 11201ES03) on the StepOnePlus Real-Time PCR System (Applied Biosystems) according to the manufacturer’s instructions. The relative gene expression was quantified using the ΔΔCt method and normalized to GAPDH. The primers used are summarized in Table S2.

**Statistical analysis**

All statistical tests were performed using GraphPad Prism version 8.0 (Graph Pad, San Diego, CA, USA). Statistical significance was determined using the Student’s t-test for experiments comparing two groups. Comparisons among groups were analyzed using analysis of variance (ANOVA). *P* values were 2-tailed and differences were determined by a statistical significance of p < 0.05, *p < 0.05, **p < 0.01, ***p < 0.001. Error bars represent SEM ± SD of three experiments unless stated otherwise.

**Table S1 The oligonucleotide sequences of genotyping PCR primers**

| Gene name | Forward (F) /Reverse (R) | Sequence (5'-3') |
| --- | --- | --- |
| PPAN-FL/WT | F | GCAACAGTCTCTTCCTGTGCCATTT |
|  | R | TCTGTTGGGGAAAGCAAGCAT |
| PPAN-Del | F | CCACCCCCTTCACTTTCTCT |
|  | R | AGGTGGCAGGTACCTTTGTG |
| Stra8-Cre | F | GTGCAAGCTGAACAACAGGA |
|  | R | CCAGCATCCACATTCTCCTT |

**Table S2 The oligonucleotide sequences of qRT-PCR primers**

| Gene name | Forward (F) /Reverse (R) | Sequence (5'-3') |
| --- | --- | --- |
| Mouse PPAN | F | GCAGCAGTTTAACCACCCTC |
|  | R | TGTTCTCCATCTGGCTCCAC |
| Mouse FBL | F | CAAAATTGAGTACAGAGCCTGGA |
|  | R | CGGGCCGACAATATCAGAGA |
| Mouse PES1 | F | TCACCCACCAGCTTGTTGAC |
|  | R | GCTTCTCAGGGGGAATGTAA |
| Mouse UBF | F | AGAACAACCTCCCATCCA |
|  | R | TACCTCGTTAGAAATCTCCAC |
| Mouse NPM1 | F | GCAGGGGCAAAAGATGAGT |
|  | R | CAAAGCCCCCTAGGGAAAC |
| Mouse 47S | F | GGTGTCCAAGTGTTCATG |
|  | R | CAAGCGAGATAGGAATGTCTTAC |
| Mouse 28S | F | CTGTCCCTACCTACTATCCA |
|  | R | CTCCCACTTATTCTACACCT |
| Mouse 18S | F | CGGCTACCACATCCAAGGAA |
|  | R | GCTGGAATTACCGCGGCT |
| Mouse 5.8S | F | CTTAGCGGTGGATCACTCGG |
|  | R | ACGCTCAGACAGGCGTAGCC |
| Mouse SINEB2 | F | ACACACCAGAAGAGGGCATC |
|  | R | GAGCACCTGACTGCTCTTCC |
| Mouse IAP | F | CCCCGTCCCTTTTTTAGGAG |
|  | R | CTCCATGTGCTCTGCCTTCC |
| Mouse MERVL | F | CTTCCATTCACAGCTGCGACTG |
|  | R | CTAGAACCACTCCTGGTACCAAC |
| Mouse LINE1 | F | AACCTACTTGGTCAGGATGGATG |
|  | R | AGTGCAGAGTTCTATCAGACCTTC |
| Mouse MinSat | F | ACTCATCTAATATGTTCTACAGTG |
|  | R | AAAACACATTCGTTGGAAACGGG |
| Mouse MajSat | F | GATTTCGTCATTTTTCAAGTCGTC |
|  | R | TTTAGAAATGTCCACTGTAGG |
| Mouse GAPDH | F | GTGCAGTGCCAGCCTCGTCC |
|  | R | CAGGCGCCCAATACGGCCAA |

**
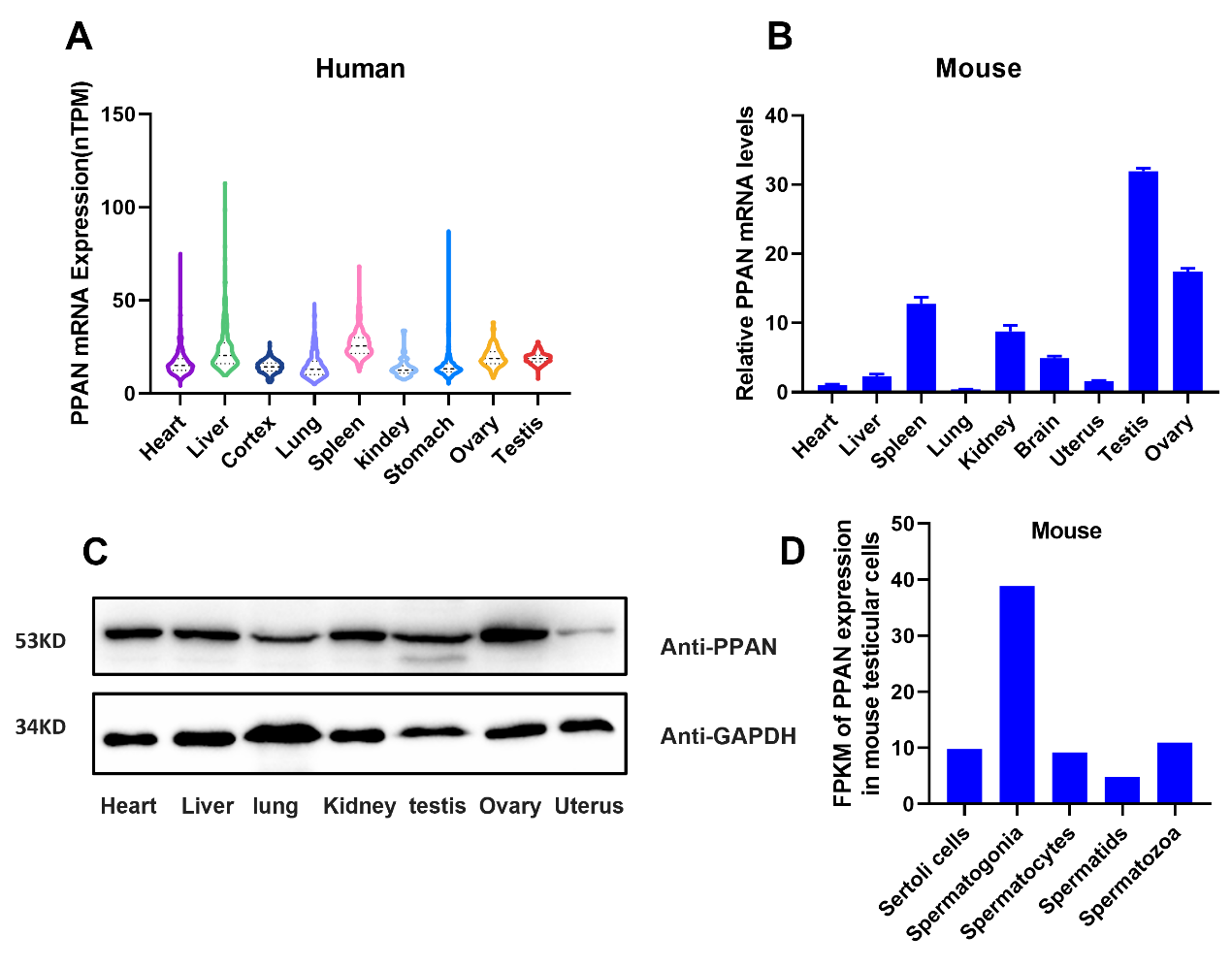
**

**Figure S1** *PPAN* is enriched in the testis with the highest expression in spermatogonium. (A) Expression analysis of *PPAN* mRNA in different human tissues (data analyzed from the Human Protein Atlas database). (B) Expression analysis of *PPAN* mRNA in different mouse tissues detected by qRT-PCR. (C) Expression of PPAN protein in different mouse tissues detected by western blot. (D) The transcription expression analysis of *PPAN* in different mouse testis cells (Data analyzed from GSE43717^1^).


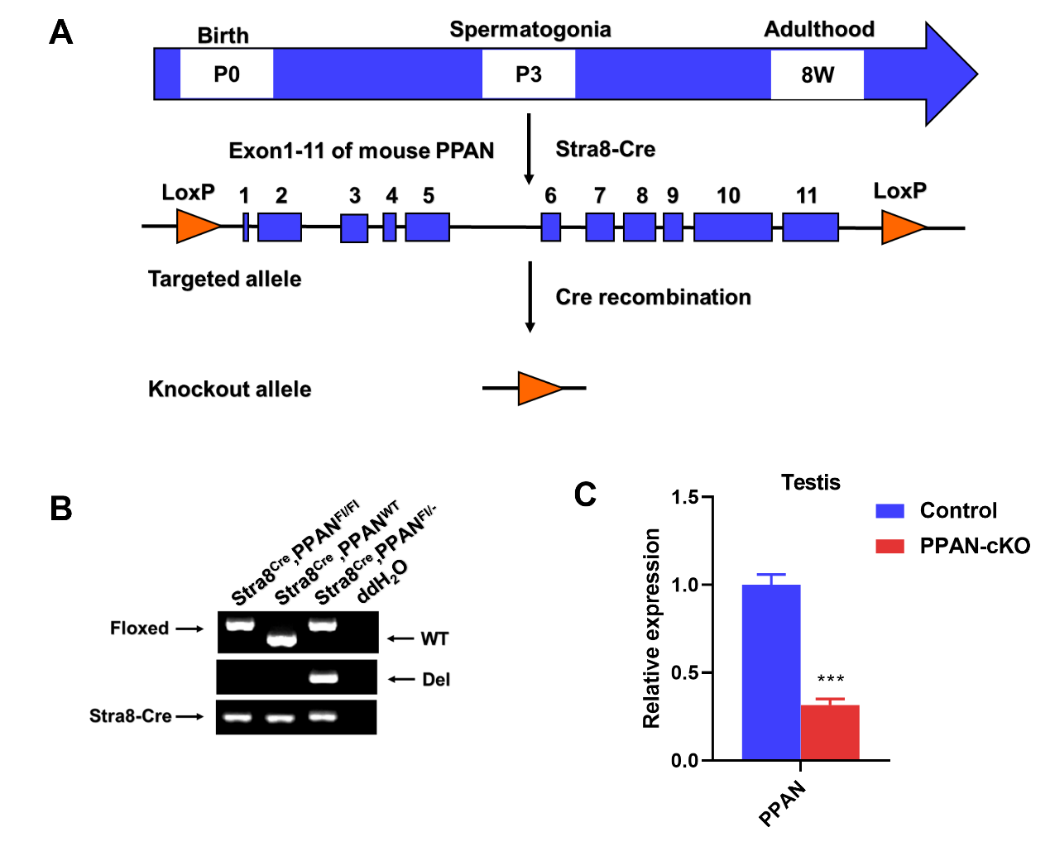


**Figure S2** Generation and validation of *PPAN*-cKO mouse model. (A) Diagram of generating *PPAN*-cKO mouse model using Stra8^Cre^ (Expression of Stra8^Cre^ initiates in type A1 spermatogonia at postnatal day 3) ^2^. (B) Representative genotyping results were visualized by agarose gel electrophoresis. (C) qRT-PCR analysis of *PPAN* mRNA in 2-month-old control and *PPAN*-cKO mouse testis.


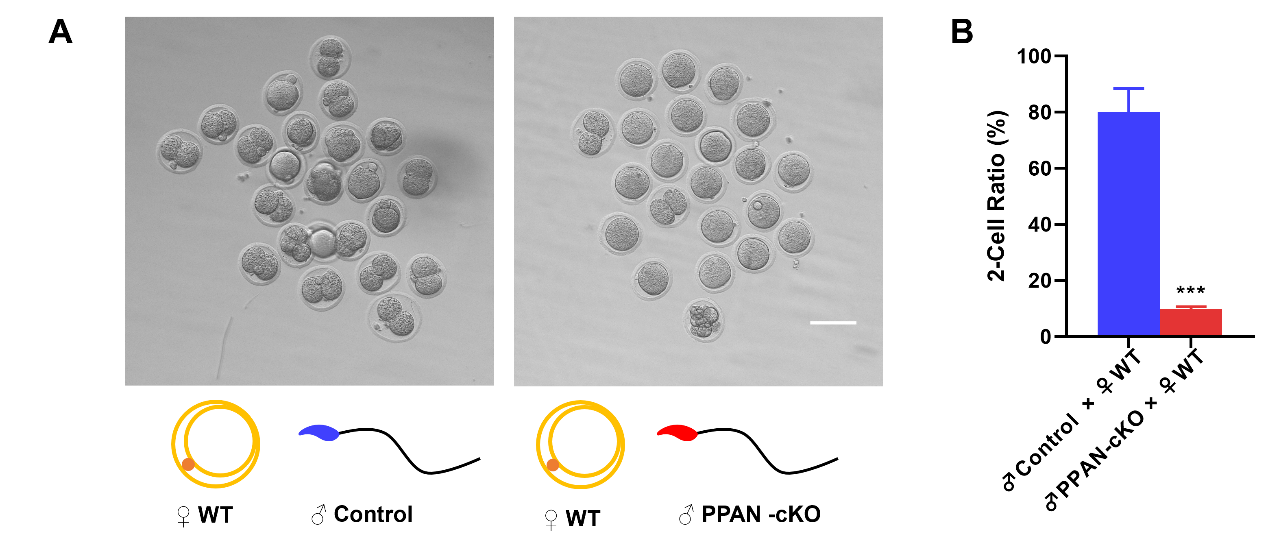


**Figure S3** Representative image of 2-Cell stage after in vitro fertilization using sperm from control and *PPAN*-cKO mice with oocytes from wildtype female mice (A), and quantitative analysis of 2-Cell rate (B) (Scale bar=100µm, n=3).

**
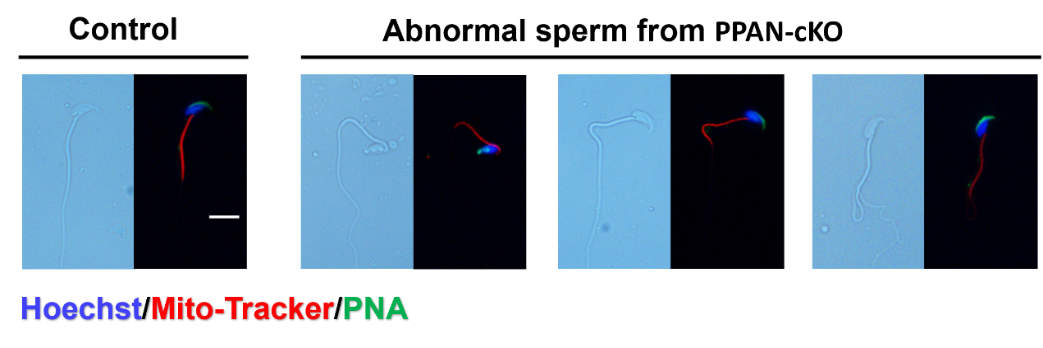
**

**Figure S4** Co-immunostaining of sperm from control and *PPAN*-cKO with Mito-Tracker for mitochondria (red), peanut agglutinin (green) for acrosome, and Hoechst (blue) for nucleus, respectively (Scale bar=10µm).


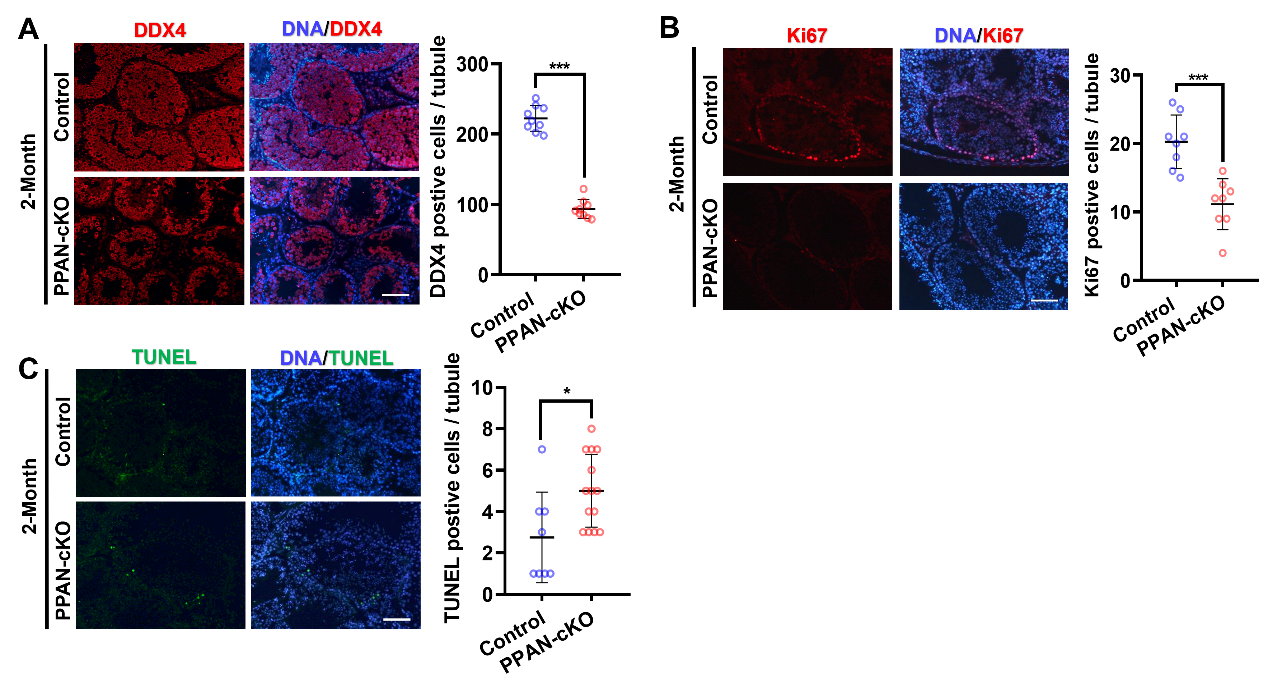


­­­­**Figure S5** The expression of DDX4 (A), Ki67 (B) and TUNEL assay (C) in seminiferous tubule frozen sections of 2-month-old control and *PPAN*-cKO by immunofluorescence and quantitative analysis (Scale bar=100μm).


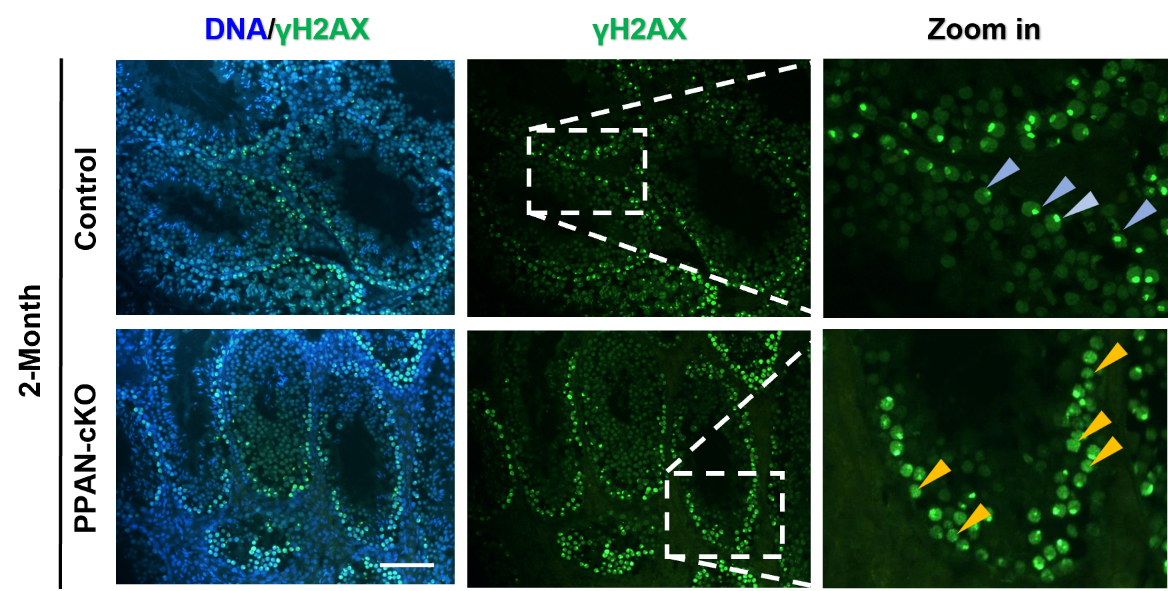


**Figure S6** Representative immunostaining image of γH2AX at stage I-VI seminiferous epithelium in 2-month-old control and *PPAN*-cKO. The predominant type of spermatocytes in the control mice is pachytene spermatocytes (indicated by gray arrows), while in the *PPAN*-cKO, there is still a significant presence of spermatocytes in the leptotene and zygotene stages (indicated by yellow arrows, scale bar=100µm)


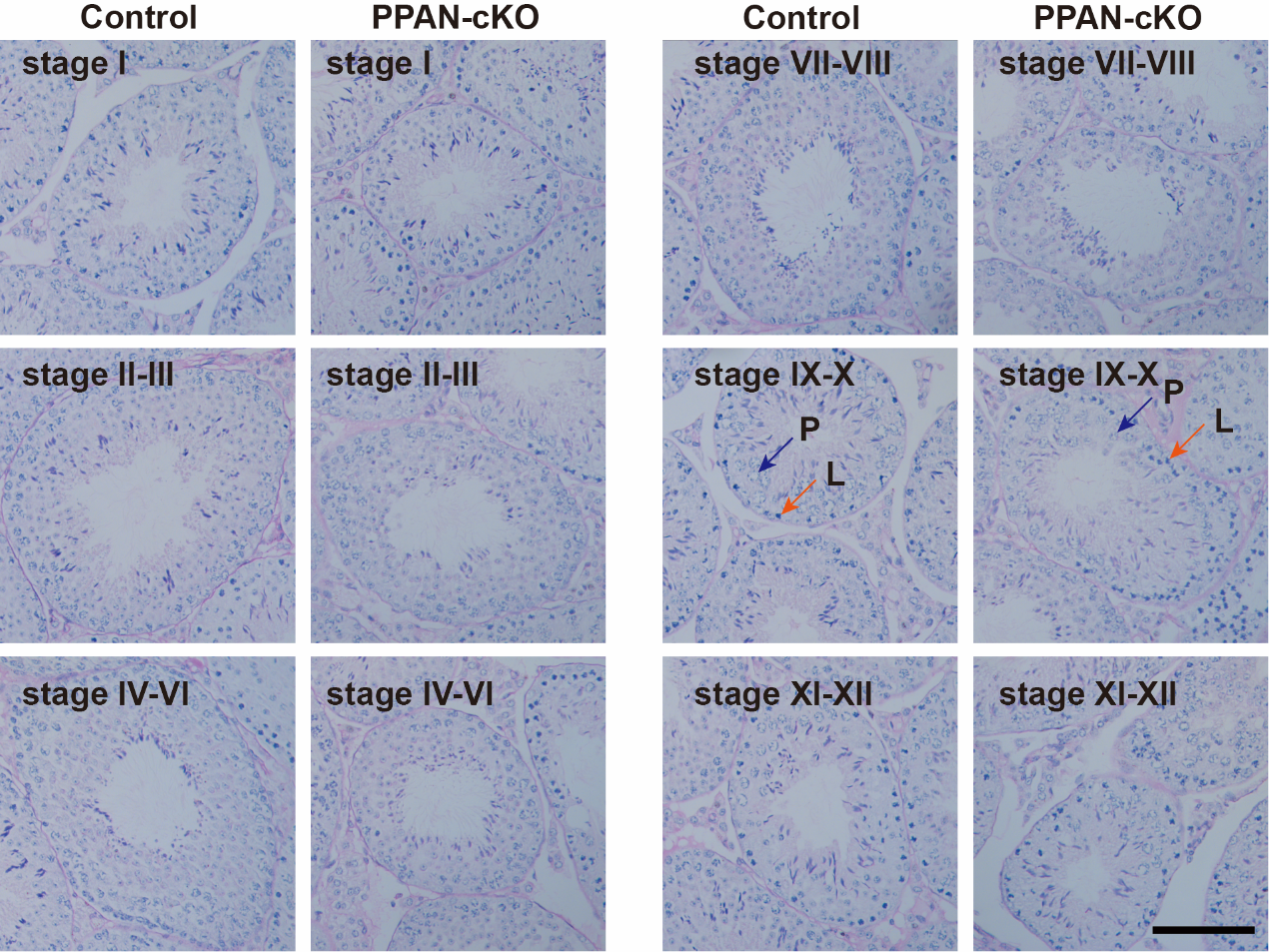


**Figure S7** Representative PAS staining of control and *PPAN*-cKO seminiferous tubules indicated a delayed transition from leptotene to pachytene stage in meiotic spermatocytes. Based on the composition of spermatocytes in the seminiferous tubules, the development of spermatids in mice is divided into 12 stages^3^, P, pachytene spermatocytes (indicated by blue arrows), L, leptotene spermatocytes (indicated by orange arrows, scale bar=100µm).


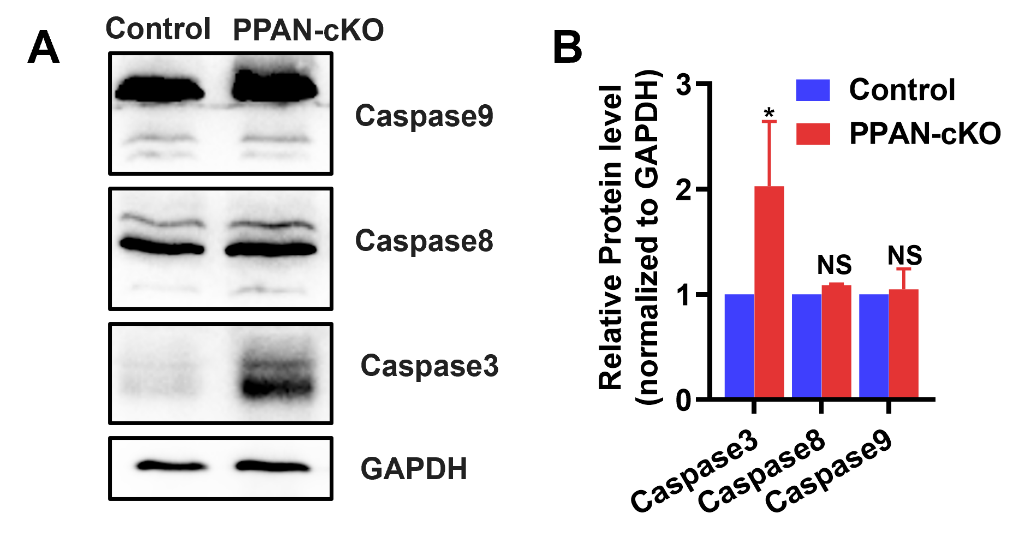


**Figure S8** Western blot analysis of proteins associated with apoptosis pathway in control and *PPAN*-cKO P10 testes (A), and quantitative analysis (B) (n=3). Caspase8/9 functions in initiation-phase of cell apoptosis, and Caspase3 functions in execution-phase of cell apoptosis.


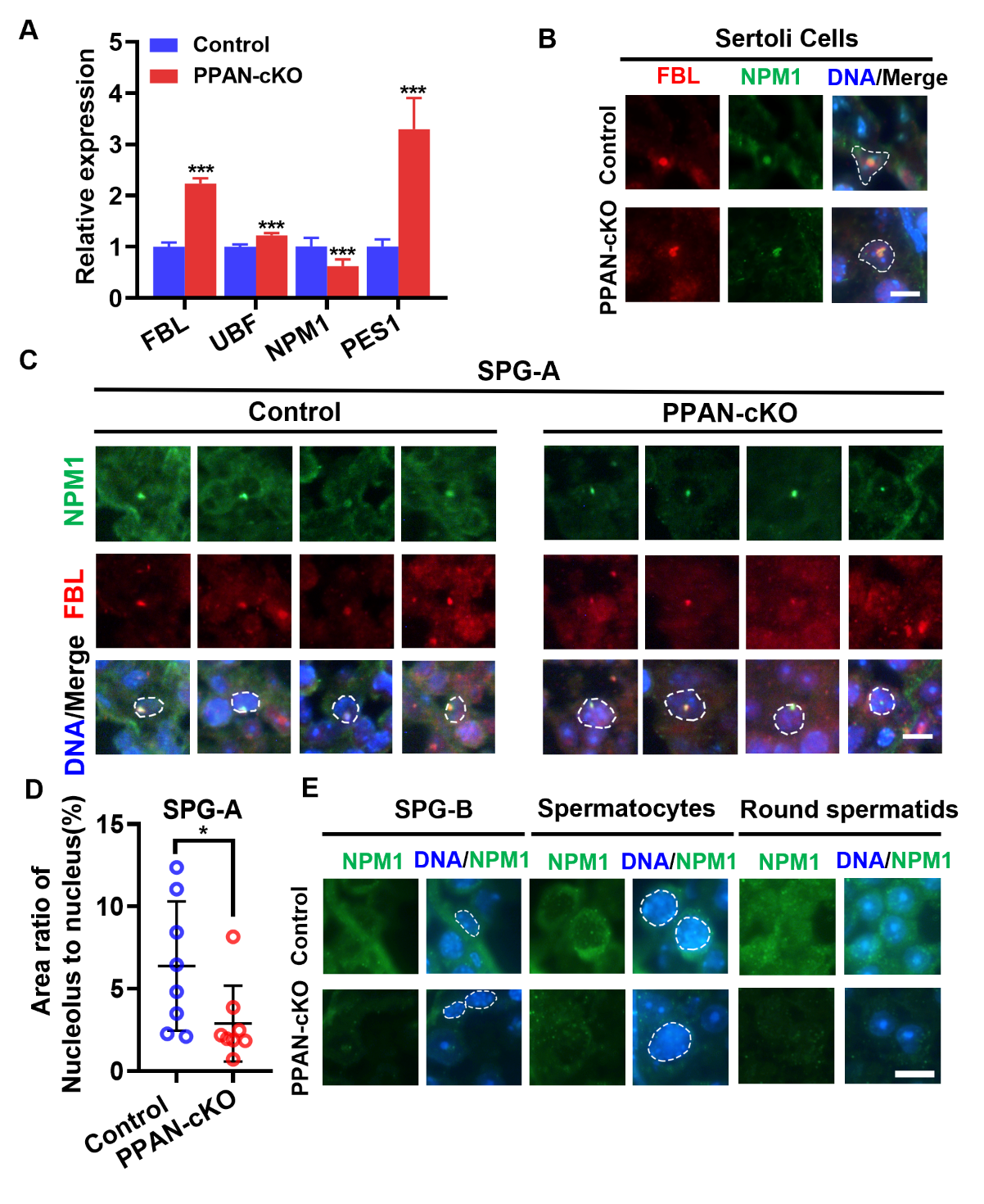


**Figure S9** Expression and localization analysis of nucleolar factors. (A) Expression changes of nucleolar factor genes in P7 testes of control and *PPAN*-cKO mice determined by qRT-PCR. (B) Typical nucleolar structure in Sertoli cells (non-germ cell) indicated by FBL and NPM1 proteins (Scale bar=10µm). (C) Representative nucleolus images in control and PPAN-cKO SPG-A indicated by FBL and NPM1 (Scale bar=10µm). Complementary to Fig. 1M. (D) Area ratio of nucleolus to nucleus in control and PPAN-cKO SPG-A, n=8. (E) Expression and location analysis of NPM1 in 2-month-old control and *PPAN*-cKO testes by immunostaining (Scale bar=10µm).

**
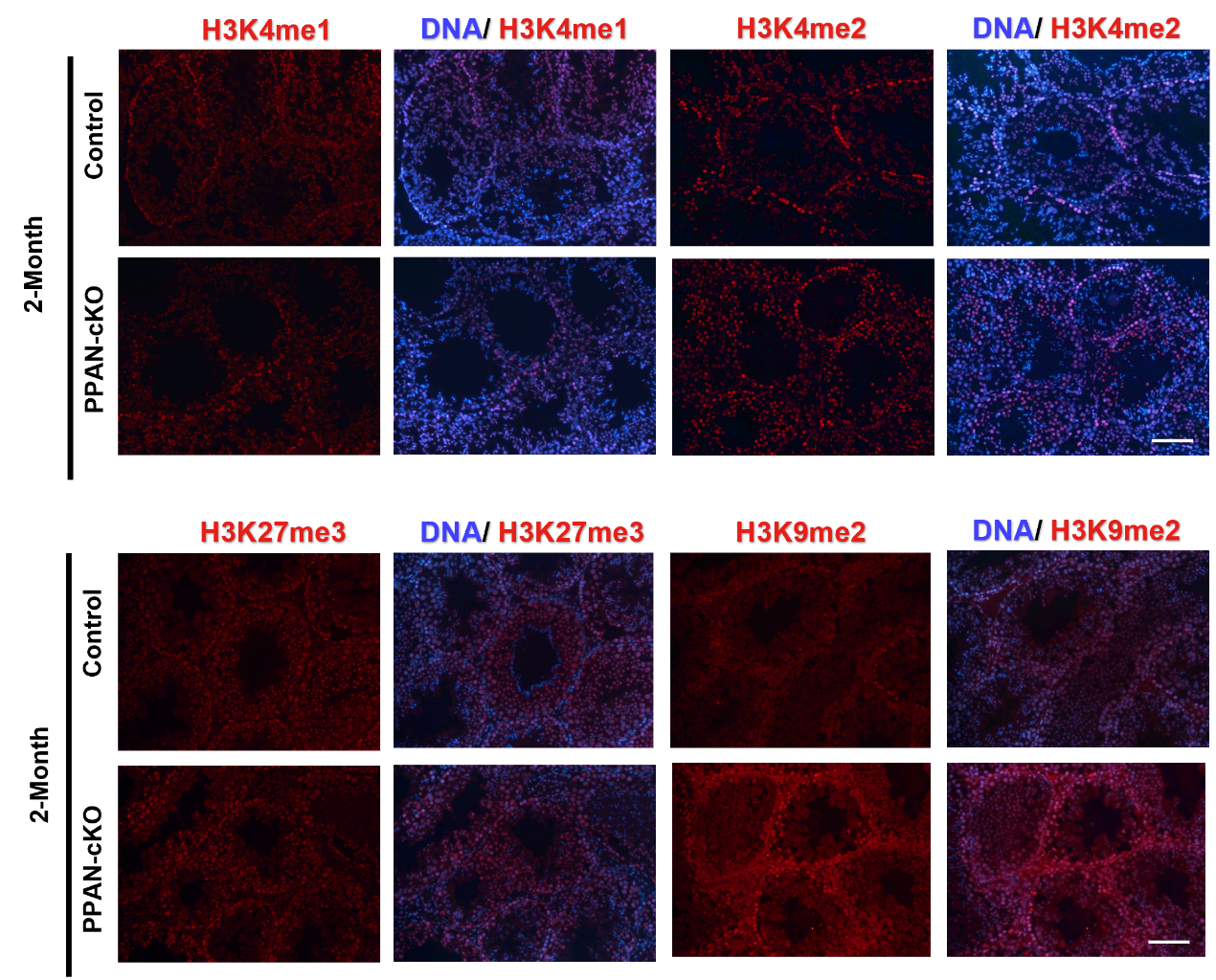
**

**Figure S10** Immunostaining of H3K4me1, H3K4me2, H3K27me3, H3K9me2 in 2-month-old control and *PPAN*-cKO testes revealed an increase in H3K9me2 levels in *PPAN*-cKO compared to control (Scale bar=100μm).

**Reference**

1. Soumillon M, Necsulea A, Weier M, et al. Cellular source and mechanisms of high transcriptome complexity in the mammalian testis. *Cell Rep.* 2013;3(6):2179-2190.

2. Sadate-Ngatchou PI, Payne CJ, Dearth AT, Braun RE. Cre recombinase activity specific to postnatal, premeiotic male germ cells in transgenic mice. *Genesis.* 2008;46(12):738-742.

3. Meistrich ML, Hess RA. Assessment of spermatogenesis through staging of seminiferous tubules. *Methods Mol Biol.* 2013;927:299-307.
